# Predictors of very early death in acute promyelocytic leukemia: a retrospective real-world cohort study

**DOI:** 10.1007/s00277-023-05422-z

**Published:** 2023-08-31

**Authors:** Joana Infante, Graça Esteves, João Raposo, João Forjaz de Lacerda

**Affiliations:** 1 Serviço de Hematologia E Transplantação de Medula, Centro Hospitalar Universitário Lisboa Norte - Hospital de Santa Maria, Lisbon, Portugal; 2 Faculdade de Medicina da Universidade de Lisboa, Lisbon, Portugal; 3 Instituto de Medicina Molecular João Lobo Antunes, Lisbon, Portugal

**Keywords:** Acute promyelocytic leukemia, Fatal bleeding, Early death, Disseminated intravascular coagulation, Coagulopathy

## Abstract

Early death (ED) is still the major obstacle to cure in acute promyelocytic leukemia (APL). Most studies focus on 30-day ED; however, little is known on predictors of death before starting APL treatment (very early death — VED) and on predictors of 7-day ED, the period with most deaths due to thrombohemorrhagic diathesis. We hypothesized whether the severity of the coagulopathy of APL could predict VED and 7-day ED. We also aimed to evaluate other characteristics associated with these outcomes. We undertook a retrospective, single-center observational study including newly diagnosed APL patients admitted to our institution between January 2000 and November 2022. Baseline demographical, clinical, and laboratorial data were collected. Statistical analysis was performed using Stata. One hundred four patients were included. The VED rate was 4.8%. A DIC Score ≥ 7 (*p* = 0.045), serum creatinine > 1.5 mg/dL (*p* < 0.001%), a DIC Score ≥ 6 within 24 h (*p* = 0.009), and mechanical ventilation (*p* < 0.001) were associated with VED. The 7-day ED rate was 12.5%. High-risk (*p* = 0.007) and hypogranular APL (*p* = 0.029), DIC Score at diagnosis (*p* = 0.047), DIC Score ≥ 7 (*p* = 0.043), DIC Score ≥ 6 within 24 h (*p* = 0.025), PT prolongation > 6 s (*p* = 0.002), and creatinine > 1.5 mg/dL (*p* = 0.004) were associated with 7-day ED. However, only elevated creatinine emerged as an independent predictor of 7-day ED (OR 21.4; *p* = 0.008). Our study shows that in patients with APL, an elevated creatinine at diagnosis strongly predicts for 7-day ED. A DIC Score ≥ 7 and a Score that remains ≥ 6 within 24 h and a serum creatinine > 1.5 mg/dL significantly associated with VED.

## Introduction

Acute promyelocytic leukemia (APL) has evolved from a grim prognosis to being the most curable subtype of acute leukemia today. Currently, the main obstacle to cure is still the significant risk of early death (ED) despite advances in targeted therapy and supportive care [1].

ED is commonly defined as death occurring within 30 days of APL diagnosis, and its incidence rate ranges from 0 to 8% in clinical trials [2, 3] to 29–32% in real-world registries [4, 5]. Even harder to estimate is the incidence of very early death (VED), occurring in newly diagnosed patients who expire before having the chance of starting all-transretinoic acid (ATRA), since these cases are underreported and excluded from clinical trials [6]. Thus, knowledge on the characteristics of these patients and on predictors of VED in APL is sorely lacking.

Most published studies in ED in APL focus on predictors of all-cause mortality during the first 30 days of APL. Bleeding is the main cause of ED and the key threat during the first week of APL diagnosis, as opposed to deaths caused by infection, differentiation syndrome, and other causes unrelated to APL’s thrombohemorrhagic diathesis, which rise beyond the first week [7]. Considering that three-quarters of all EDs occur during the first week of APL diagnosis [4], establishing the particular predictors of 7-day ED could have the most impact on refining routine care of APL patients during the hazardous first week of disease.

Since bleeding in APL is related to disseminated intravascular coagulation (DIC) and hyperfibrinolysis induced by leukemic cells, one could question whether more severe changes in coagulation parameters associate with life-threatening early bleeding and death in APL. Our group previously reported that a stable or increasing DIC Score of the International Society for Thrombosis over 48 h is associated with ED in APL patients [8].

Henceforth, we hypothesized whether the severity of the coagulopathy of APL, reflected by a higher DIC Score at diagnosis and within 24 h, could predict VED and 7-day ED. Secondly, we aimed to identify other baseline characteristics which could predict these impending catastrophic outcomes to improve current care and decrease VED and ED rates.

## Methods

We performed a retrospective, single-center observational study, in which clinical records of all APL patients consecutively admitted to our institution between January 2000 and November 2022 were collected and reviewed after our local institution’s Ethics Committee approval. Diagnosis was suspected on observation of the peripheral blood smear and prompted immediate ATRA administration if the patient was able to take the capsules. APL was later confirmed by fluorescence in situ hybridization detection of t(15;17) or by identification of a PML-RARα transcript. All patients received blood products according to international guideline recommendations [9] — fresh frozen plasma to a target INR of < 1.3, platelet transfusions if platelets < 50 × 10^9^/L and fibrinogen concentrate if serum fibrinogen < 150 mg/dL. All patients who started ATRA also began differentiation syndrome prophylaxis with prednisolone 0.5 mg/kg, as per local protocol.

Patient baseline demographics, blood work results including coagulation parameters, place of first medical evaluation, and APL characteristics were collected. The DIC Score of the International Society for Thrombosis and Hemostasis was calculated at diagnosis and up to 24 h later for each patient [10]. Very early death (VED) was defined as death occurring before initiation of APL-targeted treatment with ATRA, whereas 7-day ED was defined as death occurring within 7 days of APL diagnosis.

Statistical analysis was performed using Stata (version 13), using chi-square test and Mann–Whitney test for differences between VED versus remaining patients and between 7-day ED versus survivors beyond the first week of diagnosis. Logistic regression analysis was used to evaluate predictors of VED and of 7-day ED. A *p* value < 0.05 was considered statistically significant.

## Results

### VED, before initiation of APL treatment

A total of 104 patients were included, with a median age of 47 years (17–84). In this cohort, 5 patients died due to APL-related bleeding before starting ATRA, all within 36 h of hospital admission (VED rate of 4.8%). Regarding the type of fatal bleed, 3 patients succumbed to intracranial hemorrhage, 1 to alveolar hemorrhage, and 1 to bleeding peptic ulcers unresponsive to cauterization through upper gastrointestinal endoscopy.

The remaining 99 patients in this cohort began treatment with ATRA 45 mg/m^2^ divided in two daily doses: 82 combined with chemotherapy, 16 with arsenic trioxide and 1 as monotherapy.

Table 1 summarizes the baseline characteristics of these 5 patients. All patients who died before ATRA administration were male, aged between 32 and 67 years. High-risk APL was diagnosed in 3 patients, while 2 had intermediate-risk disease. Most patients (4 out of 5) were referred from the emergency department of non-specialized hospitals with a suspected acute leukemia, and APL was confirmed upon arrival at our institution. Two patients were already under mechanical ventilation on arrival to our center due to impaired level of consciousness caused by intracranial bleeding at disease presentation.

**Table 1:** Baseline characteristics of patients who died before receiving ATRA, including laboratory parameters of the day of APL diagnosis

Case number	1	2	3	4	5
Gender/age	M/39	M/67	M/59	M/32	M/56
Leukocytes (× 10^9^/L)	79.8	10.2	0.390	10.3	1.15
Platelets (× 10^9^/L)	16	13	13	9	10
Hemoglobin (g/dL)	7.0	11.2	8.2	9.0	9.4
Sanz risk	High	High	Intermediate	High	Intermediate
PT prolongation (seconds above control)	5.9	14.4	3.2	7.1	1.2
Fibrinogen (mg/dL)	168	132	387	207	88
d-Dimers (ng/dL)	45.2	16.5	4.2	13.9	9.1
DIC Score	6	7	5	7	6
Referred from another hospital	Yes	Yes	Yes	Yes	No
Under mechanical ventilation on arrival	Yes	No	No	Yes	No
Cause of death	Hemorrhagic transformation of ischemic stroke and disseminated thrombosis	Diffuse severe gastric bleeding	Alveolar hemorrhage	Intracranial hemorrhage	Intracranial hemorrhage
Hours between tertiary hospital admission and death	36	7	13	13	29

*DIC* Disseminated Intravascular Coagulation Score, *PT* prothrombin time

The baseline differences between the VED group and remaining patients are depicted in Table 2. The patients who died before ATRA frequently presented with a DIC Score above 7 (60.0% vs 21.2%, *p* = 0.045) and maintained a DIC Score ≥ 6 within 24 h (100% vs 49.5%; *p* = 0.027). Concomitant acute renal failure with a serum creatinine > 1.5 mg/dL was also more common (60.0% vs 7.1%, *p* < 0.001%), and VED patients were also more likely to be mechanically ventilated before arrival to our tertiary hospital (40.0% vs 0%, *p* < 0.001). There were no differences regarding age, APL risk, leukocyte blood count, and coagulation parameters between the two groups.

**Table 2:** Comparison of baseline demographical, clinical, and biological characteristics at APL diagnosis between patients with fatal bleeding before ATRA (VED) and the remaining ATRA-treated patients

	Death before ATRA (*n* = 5)	All other patients (*n* = 99)	*p*
Age, median	56 (32–67)	46 (17–84)	0.534
Male, *n*	**5 (100%)**	**47 (47.5%)**	**0.022**
Sanz risk﻿,﻿ ﻿*n*			
* Low*	0	23 (23.2%)	0.242
* Intermediate*	2 (40.0%)	48 (48.5%)	
* High*	3 (60.0%)	28 (28.3%)	
High-risk APL, *n*	3 (60.0%)	28 (28.3%)	0.130
Hypogranular APL (M3v), *n*	0 (0.0%)	7 (7.1%)	0.538
Leukocytes × 10^9^/L, median	20.3	14.0	0.699
Platelets × 10^9^/L, median	17	38	0.089
Hemoglobin (g/dL), median	9.0	9.6	0.285
DIC Score, median	6 (5–7)	5 (2–8)	0.725
DIC Score ≥ 5﻿,﻿ ﻿*n*	5 (100%)	63 (63.3%)	0.095
DIC Score ≥ 6﻿,﻿ ﻿*n*	4 (80.0%)	44 (44.4%)	0.120
DIC Score ≥ 7﻿,﻿ ﻿*n*	**3 (60.0%)**	**21 (21.2%)**	**0.045**
DIC Score ≥ 6 in the second coagulation assessment within 24 h of APL suspicion﻿,﻿ ﻿*n*	**5 (100%)**	**49 (49.5%)**	**0.027**
Prolonged PT > 3 s﻿,﻿ ﻿*n*	4 (80.0%)	55 (55.6%)	0.282
Prolonged PT > 6 s﻿,﻿ ﻿*n*	2 (40.0%)	13 (13.1%)	0.095
Fibrinogen < 100 mg/dL﻿,﻿ ﻿*n*	1 (20.0%)	22 (22.1%)	0.907
d-Dimers > 5 ng/μL﻿,﻿ ﻿*n*	4 (80.0%)	69 (70.4%)	0.645
Creatinine > 1.5 mg/dL, *n*	**3 (60.0%)**	**7 (7.1%)**	** < 0.001**
LDH (U/L), median	893	590	0.917
Under mechanical ventilation before arrival to tertiary hospital	**2 (40.0%)**	**0 (0.0%)**	** < 0.001**

*DIC* Disseminated Intravascular Coagulation Score, *PT* prothrombin time

### Early death (ED), within 7 days of admission

Among the cohort of 104 patients, a total of 13 patients died within 7 days of APL diagnosis (7-day ED rate of 12.5%). Adding to the 5 patients who died before ATRA administration, 5 patients were treated with ATRA plus idarubicin, 2 patients with 7 + 3 plus ATRA (due to initial negative FISH assay for t(15;17) but subsequent PML-RARα detection by RT-PCR), while 1 patient was intended to be treated with ATRA plus idarubicin but expired before idarubicin administration.

While all patients who succumbed before ATRA died due to bleeding, this was not the case for all the patients with 7-day ED. Bleeding was still the main cause of death (6 due to intracranial bleeding, 1 to alveolar hemorrhage, and 1 to upper gastrointestinal bleeding), whereas 2 patients died due to thrombotic complications (1 with pulmonary embolism and 1 with acute myocardial infarction) and 2 patients succumbed to sudden cardiac arrest of uncertain etiology.

Comparison of the baseline differences between the group of 7-day ED versus all other patients is depicted in Table 3. In the 7-day ED group, high-risk APL (61.5% vs 25.3%, *p* = 0.007) and hypogranular variant APL (23.1% vs 4.4%, *p* = 0.016) were significantly more frequent, median leukocyte count was higher (*p* = 0.008), and median platelet was lower (*p* = 0.028) at diagnosis. Regarding coagulation profile differences, patients dying within the first week were more likely to have a baseline DIC Score ≥ 7 (*p* = 0.035), a PT prolongation above 6 s (*p* < 0.001), and a DIC Score ≥ 6 in the second coagulation assessment within 24 h of APL suspicion (*p* = 0.011). This 7-day ED patients also had a higher LDH (*p* = 0.009) and a serum creatinine above 1.5 mg/dL (*p* = 0.001).

**Table 3:** Comparison of baseline demographical, clinical, and biological characteristics at APL diagnosis between patients with early death within 7 days of diagnosis and the remaining patients

	Death within 7 days (*n* = 13)	All other patients (*n* = 91)	*p*
Age, median	45 (26–82)	47 (18–88)	0.609
Age > 60 years, *n*	4 (30.8%)	28 (30.8%)	0.999
Male, *n*	9 (69.2%)	43 (47.3%)	0.138
Sanz risk			
* Low*	0	23 (25.3%)	0.014
* Intermediate*	5 (38.5%)	45 (49.5%)	
* High*	8 (61.5%)	23 (25.3%)	
High-risk APL, *n*	**8 (61.5%)**	**23 (25.3%)**	**0.007**
Hypogranular APL (M3v), *n*	**3 (23.1%)**	**4 (4.4%)**	**0.016**
Leukocytes × 10^9^/L, median	**10.3**	**2.1**	**0.008**
Platelets × 10^9^/L, median	**17**	**28**	**0.028**
Platelets < 50 × 10^9^/L, n	**13 (100%)**	**69 (75.8%)**	**0.046**
Hemoglobin (g/dL), median	8.6 (6.1–13.2)	9.4 (4.0–15.2)	0.256
DIC Score, median	6 (3–8)	5 (2–8)	0.079
DIC Score ≥ 5, *n*	11 (84.6%)	57 (62.6%)	0.119
DIC Score ≥ 6, *n*	8 (61.5%)	40 (44.0%)	0.234
DIC Score ≥ 7, *n*	**6 (46.2%)**	**18 (19.8%)**	**0.035**
DIC Score ≥ 6 in the second coagulation assessment within 24 h of APL suspicion﻿,﻿ *﻿n*	8 (72.7%)	20 (32.3%)	**0.011**
Prolonged PT > 3 s﻿,﻿ ﻿*n*	10 (76.9%)	49 (53.9%)	0.116
Prolonged PT > 6 s﻿,﻿* ﻿n*	**6 (46.2%)**	**9 (9.9%)**	** < 0.001**
Fibrinogen < 100 mg/dL﻿,﻿ ﻿*n*	3 (23.1%)	20 (22.0%)	0.929
d-Dimers > 5 ng/μL, *n*	10 (76.9%)	63 (70.0%)	0.608
Creatinine > 1.5 mg/dL, *n*	**5 (38.5%)**	**7 (7.7%)**	**0.001**
LDH (U/L), median	**1224**	**460**	**0.009**

*DIC* Disseminated Intravascular Coagulation Score, *PT* prothrombin time, *NA* not available

As demonstrated in Table 4A, the following factors were identified to be significantly associated with 7-day ED by univariate logistic regression: high-risk APL (OR 4.3, *p* = 0.007), hypogranular APL (OR 6.2, *p* = 0.029), DIC Score at diagnosis (OR 1.6, *p* = 0.047), DIC Score ≥ 7 (OR 3.5, *p* = 0.043), DIC Score ≥ 6 in the second coagulation assessment within 24 h of APL suspicion (OR 5.0, *p* = 0.025), PT prolongation above 6 s from control (OR 7.8, *p* = 0.002), and creatinine above 1.5 mg/dL (OR 7.5, *p* = 0.004).

**Table 4:** Univariate (A) and multivariate (B) analysis of predictors of 7-day early death (ED)

	OR	[95% CI]	*p*
(A) Univariate analysis of predictors of 7-day ED			
Sanz high risk	4.3	1.5–12.2	0.007
Hypogranular APL	6.2	1.2–31.6	0.029
DIC Score	1.6	1.00–2.5	0.047
DIC Score ≥ 7	3.5	1.04–11.6	0.043
DIC Score ≥ 6 in the second coagulation assessment within 24 h of APL suspicion	5.0	1.2–20.6	0.025
PT prolongation > 6 s	7.8	2.2–28.3	0.002
Creatinine > 1.5 mg/dL	7.5	1.9–29.2	0.004
(B) Multivariate analysis of predictors of 7-day ED			
Sanz high risk	3.1	0.4–23.2	0.278
DIC Score ≥ 7	0.8	0.1–6.4	0.874
PT prolongation > 6 s	1.6	0.2–14.1	0.683
Creatinine > 1.5 mg/dL	**21.4**	**2.2**–**205.8**	**0.008**
Hypogranular APL	7.3	0.6–90.1	0.120
DIC Score ≥ 6 in the second coagulation assessment within 24 h of APL suspicion	2.7	0.4–20.0	0.338

*DIC* disseminated intravascular coagulation, *PT* prothrombin time

Building a multivariate analysis model despite the low number of events (Table 4B), the only independent predictor of 7-day ED was a serum creatinine > 1.5 mg/dL, which confers more than 21-times increased odds of ED within 7 days (OR 21.4, *p* = 0.008).

## Discussion

This study identified an elevated baseline serum creatinine as the only independent predictor of 7-day ED, which also was significantly associated with VED. A serum creatinine > 1.5 mg/dL at diagnosis increased 21 times the odds of death within 7 days of APL diagnosis, and was present in 60% of patients with VED (versus 7.1%; *p* < 0.001).

The mechanisms behind acute renal failure in newly diagnosed APL may be several, such as microvascular thrombosis in the kidneys in the context of DIC [11], tumor lysis syndrome, or kidney hypoperfusion caused by distributive or hemorrhagic shock. Renal failure can also occur due to differentiation syndrome or to nephrotoxic drugs such as certain antibiotics, but these are frequent causes of renal failure later in APL treatment and not at diagnosis. Other published studies focusing on 30-day ED have also identified elevated serum creatinine as a marker of poorer prognosis [4, 12], so renal failure appears to be associated with worse outcomes in APL not only at diagnosis and in the early days of disease as we have shown, but also during the whole of induction treatment.

One of the hypotheses we aimed to test in this study was whether the severity of the coagulopathy of APL — which we defined as a higher DIC Score at diagnosis as well as the persistence of a high DIC Score within 24 h of disease — could predict VED and 7-day ED.

Regarding VED, while there was no statistically significant difference between the two groups in the individual coagulation baseline variables included in the DIC Score, a Score ≥ 7 was significantly associated with VED. A higher score in APL patients may thus reflect a more severe underlying coagulopathy. Additionally, the association of VED with the persistence of a high DIC Score within 24 h of disease suggests that the DIC Score may serve as a clinical tool in guiding more aggressive blood product transfusions in order to diminish the score of APL patients.

As for the impact of coagulation abnormalities on 7-day ED, a DIC Score ≥ 7 and a DIC Score ≥ 6 in the second coagulation assessment within 24 h of APL suspicion were also associated with death. Moreover, the DIC Score itself at diagnosis conferred 1.6 increased odds of 7-day ED per each 1-point Score increase, and the only coagulation parameter associated with ED was a PT prolongation above 6 s from control. These findings could suggest a potential role of these surrogates for severe coagulopathy in predicting 7-day ED, to be confirmed in a larger cohort. On multivariate analysis, only an elevated serum creatinine was significantly associated with ED.

Very few studies have focused on death before APL-specific treatment. To our knowledge, our present study and a study from Italy [13] are the only published papers focusing on predictors of death before APL treatment, which we defined as VED. In 2010, this Italian study reported 5 patients with fatal hemorrhagic complications before starting ATRA plus chemotherapy and found that these patients more frequently had delayed diagnosis, altered coagulation values, leukocytosis, a high peripheral blast count, and phenotypic expression of CD2 [13]. No logistic regression analysis was reported on this dataset. A real-life study from Brazil reported 7 patients with APL who died before receiving chemotherapy but who were given ATRA by enteric tube, who were mostly female and presented with high-risk disease, of whom 6 succumbed to intracranial or pulmonary bleeding [14].

In our cohort, the patients with VED were more likely to have altered coagulation values (as reflected by a DIC Score above 7 and a DIC Score ≥ 6 within 24 h of diagnosis), elevated creatinine, and to be mechanically ventilated before arrival to our center.

The fatal cases in our cohort reflect the difficulty of treating APL patients already in a critically ill condition upon hospital arrival due to a catastrophic disease presentation, which not only excludes their participation in trials but also does not allow delivery of standard APL therapy. Urgent treatment with ATRA is paramount upon APL suspicion since it counteracts the biochemical profile of coagulopathy-inducing atypical promyelocytes [15]. However, treatment with ATRA may be hindered for several reasons. First, patients may be unable to swallow capsules due to decreased alertness, because of intracranial bleeding, multiorgan dysfunction, or sedation for mechanical ventilation. In these scenarios, ATRA could be given via a nasogastric tube placed with precaution, but the size of the capsules and the inability to crush them can hamper their nasogastric administration. There are anecdotal reports on heating ATRA capsules in water until they melt [16], or dissolving ATRA with water and mineral oil into a slurry which can be more easily swallowed or administered via the tube [17]. The second scenario is that of patients under *nil per os* because of active gastric bleeding. These rare situations constitute a need for a commercialized intravenous formulation of ATRA, which has been studied previously as a liposomal formulation that was effective in inducing APL complete remission [18].

In our institution’s cohort, the mortality rate before starting treatment was 4.8%, which is comparable to that reported in the previously cited Italian single-center study (4.7%) [13]. In a Swedish population study, among the 29% of patients who died within 30 days of APL diagnosis, 35% never received ATRA, yielding a VED rate of approximately 10.5% [12]. These numbers show that death before APL treatment is frequent, underreported and probably underestimated, and preventing VED represents one of the few unmet needs in the field of APL.

In conclusion, while most ED studies in APL focus on 30-day mortality, our study shed light on predictors of VED and 7-day ED which are mediated mostly by the thrombohemorrhagic diathesis of APL. We identified a serum creatinine > 1.5 mg/dL, DIC Score ≥ 7, and a DIC Score ≥ 6 in the second coagulation assessment within 24 h of APL suspicion as being significantly associated with VED (with the statistical limitation of a small number of deaths defined as VED). High-risk APL, increasing DIC Score at diagnosis, a DIC Score ≥ 7, a DIC Score ≥ 6 in the second coagulation assessment within 24 h of APL suspicion, PT prolongation above 6 s from control, creatinine above 1.5 mg/dL, and hypogranular APL were identified as predictors of 7-day ED, with elevated creatinine emerging as the sole independent prognostic factor for death.

We highlight these potential prognostic factors to aid clinicians in recognizing patients with high risk of VED and 7-day ED, particularly an elevated baseline serum creatinine which is the only independent predictor of 7-day ED. The presence of these factors can contribute to considering alternative ways to deliver ATRA in unstable patients, and to intensifying renal support and disrupted hemostasis correction through intensive blood product support, in the hopes of overcoming one of the remaining obstacles to cure in APL.

## Data Availability

The datasets generated during the current study are available from the corresponding author on reasonable request.
